# Square dance as a cultural practice: a mediating pathway of social participation toward well-being in older adults

**DOI:** 10.3389/fpubh.2026.1799066

**Published:** 2026-04-28

**Authors:** Keying Song, Zijian Zhao, Jinjin Zhang, Jianye Li, Mariusz Lipowski

**Affiliations:** 1 Gdansk University of Physical Education and Sport, Gdansk, Poland; 2 Zhengzhou University, Zhengzhou, China; 3 Zhengzhou Technology and Business University, Zhengzhou, China; 4 Shanxi Medical University, Jinzhong, China; 5 WSB Merito University Gdansk, Gdansk, Poland

**Keywords:** aged, dance therapy, depression, physical activity, psychological well-being, social participation

## Abstract

**Background:**

Population aging poses significant challenges to psychological well-being, with depression and social isolation highly prevalent among older adults. Square dance (guangchangwu), a culturally embedded group physical activity widely practiced in China, has been associated with mental health benefits; however, the mechanisms underlying these effects remain insufficiently understood. This study examined whether social participation mediates the relationship between square dance participation and well-being outcomes among older adults.

**Methods:**

A 12-week, community-based, single-blind randomized controlled trial was conducted with 150 community-dwelling adults aged 60–79 years in Zhengzhou, China. Participants were randomly assigned to a square dance intervention group (*n* = 75) or a health education control group (*n* = 75). Social participation, subjective well-being (WHO-5), and depressive symptoms (GDS-15) were assessed at baseline and post-intervention. Mediation analyses were performed using PROCESS Model 4 with bootstrapping (5,000 samples), adjusting for demographic and health-related covariates.

**Results:**

Compared with controls, the square dance group showed significantly greater improvements in social participation (Δ = +3.28), subjective well-being (Δ = +5.73), and reductions in depressive symptoms (Δ = −3.85) (all *p* < 0.001). Mediation analyses demonstrated that social participation significantly mediated the effects of square dance on both well-being (indirect effect = 2.92, 95% CI ≠ 0) and depressive symptoms (indirect effect = −2.82, 95% CI ≠ 0), indicating partial mediation.

**Discussion:**

Square dance participation significantly enhanced psychological well-being and reduced depressive symptoms among older adults, with social participation statistically accounting for part of the intervention effect. However, because mediator and outcomes were assessed at the same post-intervention time point, causal ordering cannot be definitively established. These findings highlight the importance of culturally meaningful, group-based physical activities as scalable strategies for promoting mental health and social integration in aging populations.

## 1 Introduction

Global population aging presents unprecedented public health challenges, with individuals aged 60 years and above projected to double from 12 to 22% between 2015 and 2050 (World Health Organization, 2022). China faces particularly rapid aging, with over 280 million older adults currently representing 19.8% of the population (National Bureau of Statistics of China, 2023). This demographic shift necessitates urgent strategies promoting healthy aging, functional independence, and quality of life. Aging involves physiological, psychological, and social changes significantly impacting well-being. Older adults experience declines in physical function, increasing fall risk and dependency (1), alongside heightened vulnerability to depression, anxiety, and loneliness (2). Depression affects 10–20% of community-dwelling older adults, contributing to increased morbidity, functional impairment, and mortality (3). Social isolation represents an equally critical concern, with effects comparable to smoking and obesity (4). Social participation, involvement in community activities providing interaction, emerges as a critical determinant of successful aging, associated with better physical health, cognitive function, psychological well-being, and reduced mortality (5).

Regular physical activity represents a cornerstone of healthy aging, with extensive benefits across physical, cognitive, and psychosocial domains (6). The WHO recommends older adults accumulate at least 150 min of moderate-intensity aerobic activity weekly. Physical activity reduces depression risk with effect sizes comparable to pharmacological interventions (7), operating through neurobiological pathways including enhanced neuroplasticity, increased BDNF expression, and reduced inflammation (8). Despite these benefits, approximately 25% of adults worldwide fail to meet recommended activity levels, with higher inactivity among older groups (9). Group-based physical activities offer advantages over individual exercise, including enhanced social support, peer modeling, motivation, and sense of belonging (10). Evidence indicates superior adherence rates and additional benefits for social connection and mental health compared to individual programs (11). Dance interventions particularly demonstrate benefits across physical fitness, cognitive function, psychological well-being, and social outcomes (12, 13).

Square dance (guangchangwu) represents a distinctive cultural phenomenon in contemporary China, emerging as one of the most popular recreational activities among middle-aged and older adult. Involving synchronized group routines in public spaces, square dance embodies grassroots community initiative, reflecting collective action values and transforming urban spaces into sites of social interaction. Participants are predominantly female (70–90%), middle-aged to older adults with diverse socioeconomic backgrounds (14).

Emerging research documents square dance benefits including improved cardiovascular fitness, muscular strength, balance, and flexibility (15). Mental health studies show associations with higher life satisfaction and well-being, with interventions demonstrating reduced depression and improved quality of life. Cognitive benefits have also been reported (16). Despite accumulating evidence, mechanisms underlying square dance benefits remain poorly understood. Social participation represents a plausible mediating pathway, as square dance involves coordinated action, shared objectives, and social relationship development. Theoretical perspectives suggest social participation enhances well-being through social integration, network development, emotional support, reduced loneliness, and enhanced self-esteem (17, 18). Empirical evidence supports social factors mediating relationships between group exercise and health outcomes (19). However, formal mediation analysis of square dance pathways remains absent, representing a critical research gap.

Mediation analysis examines mechanisms through which interventions exert effects, identifying intermediate variables on causal pathways (20). Understanding mediating mechanisms has implications for theory development, intervention refinement, and translational application. The present randomized controlled trial addresses this gap by examining effects of a 12-week square dance intervention on well-being and depression among Chinese older adults, specifically testing social participation as a mediating mechanism. This research advances mechanistic understanding of group-based physical activity interventions, provides experimental evidence consistent with a mediating role of social participation in the association between square dance participation and well-being, addresses culturally relevant health promotion, and contributes to positive psychology perspectives emphasizing flourishing in later life (21).

This study addresses a critical gap in healthy aging research by examining social participation as a mediating mechanism linking square dance to well-being among older adults. While previous studies have documented health benefits of square dance, none have employed rigorous mediation analysis to understand *how* these benefits occur. With China’s rapidly aging population exceeding 280 million individuals aged 60+ (National Bureau of Statistics of China, 2023), identifying scalable, culturally appropriate interventions is urgent. Square dance represents grassroots, cost-effective community practice requiring minimal infrastructure, making it highly sustainable and replicable across diverse settings. Despite widespread square dance participation in China, three critical gaps persist: (1) limited randomized controlled trials establishing causal effects on psychological well-being, (2) insufficient understanding of underlying mechanisms, particularly social pathways, and (3) absence of formal mediation analyses testing theoretical models. Existing research predominantly employs cross-sectional designs limiting causal inference.

## 2 Materials and methods

### 2.1 Participants of the study

#### 2.1.1 Sample size calculation

Sample size was calculated based on anticipated improvements in the primary outcome measure. Assuming a medium effect size (Cohen’s *d* = 0.50) for between-group differences, with a two-tailed alpha of 0.05 and statistical power of 80%, a minimum of 64 participants per group was required. Accounting for an anticipated dropout rate of 15%, the target enrollment was set at 75 participants per group, yielding a total sample of 150 participants.

#### 2.1.2 Recruitment

Older adults were recruited through multiple community-based channels in Zhengzhou, Henan Province, China, including older adults activity centers, community health service stations, residential bulletin boards, neighborhood posters, and local older adults WeChat groups. Community health workers facilitated initial contact, distributed study information, and assisted with preliminary eligibility screening. Interested individuals attended an information session where the study purpose, procedures, potential risks and benefits, and rights of participants were explained. Those willing to participate underwent formal eligibility screening.

#### 2.1.3 Eligibility criteria

*Inclusion criteria*: Participants were eligible for inclusion if they met all of the following criteria: aged 60–79 years at the time of enrollment; community-dwelling residents of Zhengzhou with an expectation to remain in the local area for the duration of the study (≥3 months); able to ambulate independently without the use of assistive devices (e.g., canes, walkers, wheelchairs); had not participated in structured square dance or any organized group exercise program more than twice per week during the preceding 3 months; demonstrated adequate cognitive function, defined as a Mini-Mental State Examination (MMSE) score ≥24; and willing and able to provide written informed consent.

*Exclusion criteria*: Participants were excluded if they met any of the following criteria: presence of severe cardiovascular or cerebrovascular disease, including recent myocardial infarction (within the past 6 months), stroke (within the past 6 months), unstable angina, or uncontrolled hypertension (systolic blood pressure ≥180 mmHg or diastolic blood pressure ≥110 mmHg); severe musculoskeletal disorders (e.g., severe osteoarthritis, recent fractures, joint replacement within the past 6 months) that would preclude safe participation in moderate-intensity physical activity; diagnosed neurological conditions (e.g., Parkinson’s disease, severe peripheral neuropathy) or psychiatric disorders (e.g., major depressive disorder with active symptoms, schizophrenia) that would impair the ability to follow instructions or cooperate with study procedures; current enrollment in another clinical intervention trial; or any other medical condition deemed by a community physician to contraindicate participation in group exercise.

#### 2.1.4 Baseline characteristics

Consistent with the demographic profile of typical square dance participants in China, the study sample was predominantly female (target: approximately 75–80%). Baseline demographic and health-related variables collected included age, sex, education level (primary school, junior high school, high school, college and above), marital status (married, widowed, single), living arrangement (living alone vs. living with family), number of chronic conditions (0, 1, ≥2), and body mass index (BMI).

### 2.2 Study design

This study employed a 12-week, community-based, single-blind, two-arm randomized controlled trial (RCT) design. The trial was conducted in Zhengzhou, Henan Province, China, from three urban residential communities with established older adults activity centers. Participants were randomly assigned in a 1:1 ratio to either a Square Dance Intervention Group or a Health Education Control Group. The study was designed in accordance with the Consolidated Standards of Reporting Trials (CONSORT) guidelines for randomized trials and utilized a mediation mechanism model to examine the pathway through which square dance participation (*X*) influences well-being (*Y*) through social participation (*M*). Ethical approval was obtained from the Institutional Review Board of Zhengzhou Technology and Business University, China (Approval No.: IEC/ZTBU/SPE/759/2025-26). All participants provided written informed consent prior to enrollment.

### 2.3 Sampling technique and sample size

A convenience sampling technique was employed through community-based recruitment channels. Participants were recruited from three urban residential communities in Zhengzhou, Henan Province, with the assistance of community health workers and older adults activity centers. The total sample size of 150 participants (*n* = 75 per group) was determined based on power analysis to detect medium effect sizes (Cohen’s *d* = 0.50) in the mediation model with adequate statistical power (*β* = 0.80, *α* = 0.05), accounting for a 15% anticipated dropout rate.

### 2.4 Randomization and allocation concealment

Randomization was performed after the completion of all baseline assessments. An independent biostatistician, who had no involvement in participant recruitment or outcome assessment, generated the randomization sequence using a computer-based random number generator. To ensure concealment, group assignments were placed in sequentially numbered, sealed, opaque envelopes prepared by an independent third party. Envelopes were opened only after participants had completed baseline assessments and confirmed enrollment, at which point group allocation was revealed to the intervention coordinator. The randomization sequence was inaccessible to outcome assessors and data analysts to maintain allocation concealment throughout the study.

### 2.5 Blinding

Due to the behavioral nature of the intervention, participants and intervention facilitators could not be blinded to group assignment. However, outcome assessments were conducted by research assistants who were not involved in intervention delivery and were unaware of group allocation. Participants were explicitly instructed not to disclose their group status during assessments. Data analysts were provided with anonymized datasets containing coded group labels. Although efforts were made to maintain assessor blinding, complete concealment cannot be guaranteed in community-based behavioral trials.

### 2.6 Training protocol

#### 2.6.1 Intervention group: square dance program

Participants in the intervention group engaged in a structured 12-week square dance program conducted three times per week. Each session lasted 60 min and was delivered in a community activity center under instructor supervision.

Each session consisted of:

- 10-min warm-up including light stretching and low-intensity rhythmic movements
- 40-min square dance practice
- 10-min cool-down with breathing exercises and static stretching

The square dance routines were standardized and included commonly practiced Chinese community square dance sequences characterized by repetitive rhythmic step patterns and coordinated upper-limb movements. Music used during sessions consisted of popular Chinese square dance tracks with a tempo ranging from 100 to 120 beats per minute, progressing gradually in complexity and coordination demands. Weeks 1–4 focused on basic step patterns and familiarization with movement sequences. Weeks 5–8 introduced increased coordination complexity and directional changes. Weeks 9–12 emphasized sustained rhythmic performance and improved synchronization.

Sessions were led by a certified community square dance instructor with more than 6 years of experience in group-based exercise instruction. Intervention fidelity was maintained using a standardized session plan and periodic supervision by the research team. Attendance was recorded at each session. Participants attending at least 80% of sessions were considered adherent to the intervention protocol (see Table 1).

**Table 1:** Weekly progression of the 12-week square dance training protocol.

Week	Training phase	Intensity (RPE)	Content of square dance training
1	Familiarization and adaptation	10-11 (low–moderate)	Introduction to square dance; slow rhythmic walking; basic stepping patterns; simple arm movements; emphasis on posture, coordination, and group interaction
2	Familiarization and adaptation	10-11 (low–moderate)	Repetition of basic steps; gentle directional changes; synchronized movements with slow-tempo music; development of movement confidence
3	Familiarization and adaptation	10-11 (low–moderate)	Gradual increase in continuous dance duration (5–7 min bouts); coordination of upper and lower limbs; enhanced group synchronization
4	Familiarization and adaptation	11 (moderate)	Consolidation of learned routines; improved rhythm control; short group formations to promote social interaction
5	Skill development and endurance	11-12 (moderate)	Introduction of new step sequences; moderate-tempo music; increased movement variety; continuous dance bouts of 8–10 min
6	Skill development and endurance	11-12 (moderate)	Directional changes and rhythm variation; balance-oriented movements; partner and group-based dance formations
7	Skill development and endurance	12 (moderate)	Longer continuous dance sequences (10–12 min); improved coordination and aerobic endurance; emphasis on smooth transitions
8	Skill development and endurance	12-13 (moderate)	Combination of previously learned routines; increased tolerance to tempo; enhanced group cohesion and enjoyment
9	Consolidation and optimization	12-13 (moderate)	Sustained square dance sequences (12–15 min); coordinated upper- and lower-body movements; emphasis on fluency and rhythm
10	Consolidation and optimization	12-13 (moderate)	Performance-style group routines; improved synchronization; maintenance of moderate aerobic intensity
11	Consolidation and optimization	12-13 (moderate)	Continuous group dance sessions; focus on mastery, confidence, and social bonding
12	Consolidation and optimization	12-13 (moderate)	Final consolidation of routines; enjoyable group performance; reinforcement of long-term physical activity adherence

Sessions were conducted three times per week, with each session lasting 60 min (10-min warm-up, 40-min main square dance activity, and 10-min cool-down). Exercise intensity was monitored using the Borg Rating of Perceived Exertion (RPE) scale. The training protocol aligns with World Health Organization physical activity guidelines for older adults.

#### 2.6.2 Control group: health education

The control group received standard health education materials on topics related to healthy aging but did not participate in the square dance intervention or any structured group exercise program. This design allowed for comparison while ensuring the control group received some benefit from study participation.

**Figure 1:**
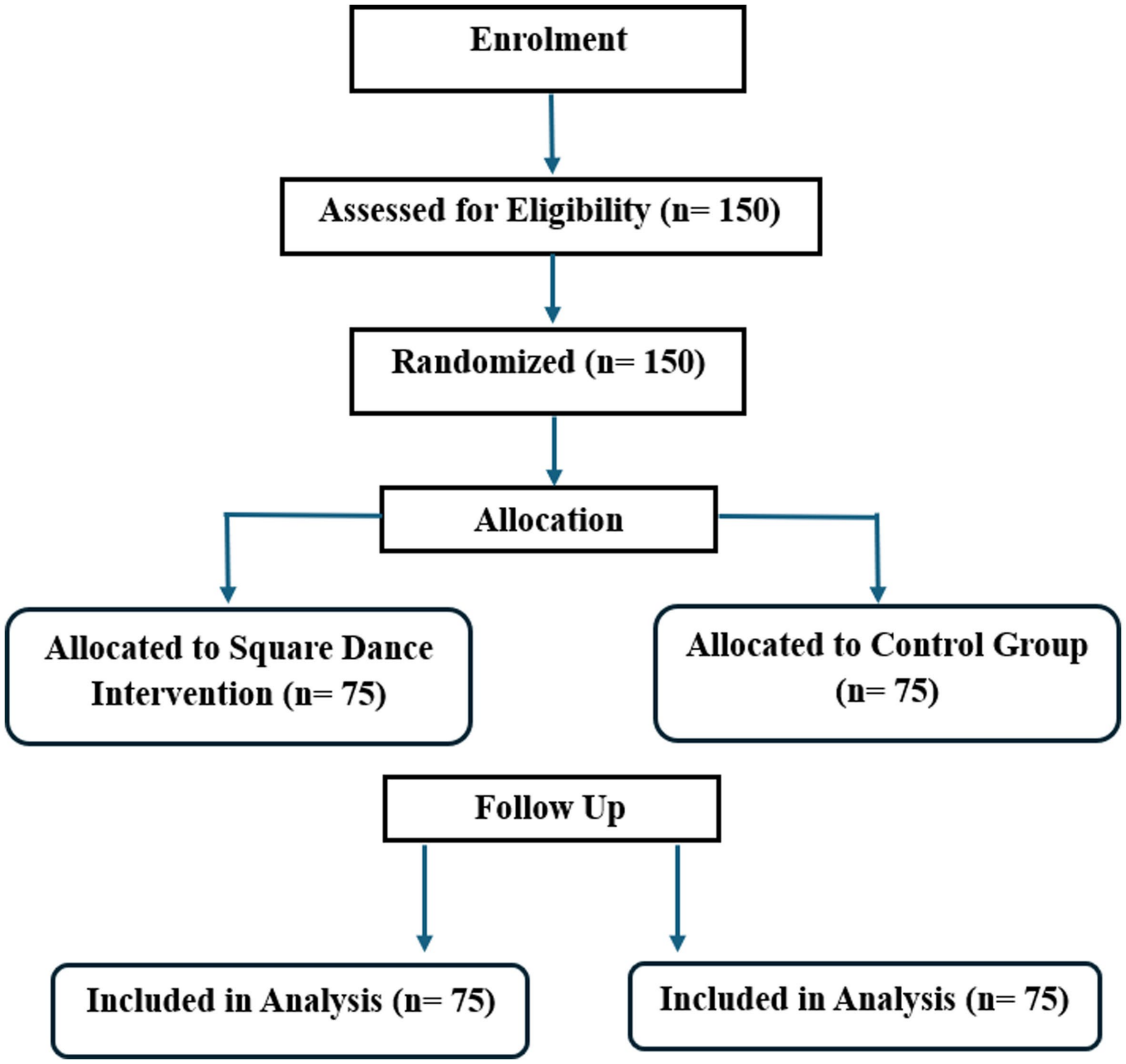
CONSORT flow diagram of participant recruitment, allocation, follow-up, and analysis.

The participant flow through the trial is illustrated in Figure 1. A total of 150 older adults were assessed for eligibility and subsequently randomized into either the square dance intervention group (*n* = 75) or the control group (*n* = 75). All randomized participants received their allocated condition and completed the 12-week study period. No participants were lost to follow-up, and all 150 participants were included in the final intention-to-treat analysis.

### 2.7 Outcome measurements

#### 2.7.1 Assessment schedule

Outcome and mediator variables were assessed at baseline (Week 0) and immediately after completion of the 12-week intervention (Week 12). All assessments were performed by trained research assistants who were blinded to group allocation.

#### 2.7.2 Independent variable

*Square dance participation (X)*: Operationalized as a binary variable indicating group assignment (0 = Control Group, 1 = Intervention Group). This variable represented the primary independent variable in the mediation model, examining whether square dance participation increases social participation (*X* → *M*) and well-being (*X* → *Y*).

*Intervention compliance/participation intensity (dosage variable)*: Attendance was recorded as a continuous variable representing the actual number of sessions attended (0–36) or proportionally calculated as the number of sessions attended divided by the number of sessions expected. This variable was included for sensitivity analyses to examine dose–response relationships, testing whether higher attendance was associated with greater improvements in social participation and well-being.

#### 2.7.3 Mediating variable

*Social participation (M)*: Social participation was assessed using a structured 5-item questionnaire evaluating the frequency of involvement in community and social activities, including group exercise participation, community organizations, interest groups, neighborhood gatherings, and volunteer engagement. Each item was rated on a 5-point Likert scale ranging from 1 (almost never) to 5 (very frequently). Total scores were calculated by summing item responses, yielding a possible range from 5 to 25, with higher scores indicating greater social participation.

The scale demonstrated acceptable internal consistency in the present sample (Cronbach’s *α* = 0.82). Previous research has supported the construct validity of similar brief social participation measures in older adult populations. In the current study, measurements were obtained at baseline and post-intervention.

#### 2.7.4 Outcome variables

The primary outcomes of interest were subjective well-being (WHO-5) and depressive symptoms (GDS-15). Other physical and mental health measures were considered secondary exploratory outcomes and are reported to provide broader context regarding intervention effects.

Primary outcome—subjective well-being (Y1): Assessed using the World Health Organization Well-Being Index (WHO-5), a validated 5-item scale measuring positive psychological well-being. Scores range from 0 to 25, with higher scores indicating better well-being. Measurements were obtained at both T0 and T1. The analysis variable was calculated as change in well-being: ΔWB = WB_T1 - WB_T0, where positive values indicate increased well-being.

Secondary outcome—depressive symptoms (Y2): Evaluated using the 15-item Geriatric Depression Scale (GDS-15), a validated self-report instrument for assessing depressive symptomatology in older adults. Scores range from 0 to 15, with higher scores indicating greater depressive symptoms. Measurements were obtained at T0 and T1. The analysis variable was calculated as change in depression: ΔDEP = DEP_T1 – DEP_T0, where negative values indicate reduced depression.

#### 2.7.5 Covariates

To control for potential confounding variables and ensure the mediation model was appropriately adjusted, the following demographic and health-related covariates were assessed at baseline: age (continuous variable in years), sex (male/female), education level (categorized as primary school, junior high school, high school, college and above), marital status (married, widowed, single), living arrangement (living alone vs. living with family), number of chronic conditions (0, 1, ≥2), baseline physical activity level (optional), and baseline scores of the mediating and outcome variables (SP_T0, WB_T0, DEP_T0).

#### 2.7.6 Additional health measures

*Physical health measures*: Aerobic endurance was assessed using the 6-Minute Walk Test (6MWT); balance was evaluated using the Timed Up and Go (TUG) test; flexibility was measured using the Chair Sit-and-Reach Test; and resting systolic and diastolic blood pressure were measured using a validated automated sphygmomanometer after 5 min of seated rest.

*Additional mental health measures*: Anxiety symptoms were evaluated using the Generalized Anxiety Disorder 7-item scale (GAD-7), with scores ranging from 0 to 21. Health-related quality of life was assessed using the 12-Item Short Form Health Survey (SF-12), yielding Physical Component Summary (PCS) and Mental Component Summary (MCS) scores.

#### 2.7.7 Process measures and adverse events

Attendance records, participant satisfaction questionnaires, and qualitative feedback were collected to evaluate intervention fidelity and acceptability. All adverse events occurring during the study period were documented, including the nature, severity, and relationship to the intervention. Serious adverse events were reported to the ethics committee within 24 h.

### 2.8 Statistical technique

All data were collected using standardized case report forms and entered into a secure, password-protected electronic database with double data entry to minimize transcription errors, with regular data quality checks performed in accordance with institutional data protection policies. Descriptive statistics were computed for all variables, including means and standard deviations for continuous variables, and frequencies and percentages for categorical variables, while independent samples *t*-tests and Chi-square tests were used to examine baseline equivalence between intervention and control groups across demographic characteristics and outcome measures. The primary analysis employed mediation modeling to test the hypothesized pathway: Square dance participation (*X*) → Social participation (*M*) → Well-being/depression (*Y*), with two separate mediation models conducted: (1) *X* → *M* → ΔWB examining subjective well-being measured by the World Health Organization Well-Being Index (WHO-5) (22) as the primary outcome, and (2) *X* → *M* → ΔDEP examining depression measured by the 15-item Geriatric Depression Scale (GDS-15) (23) as the secondary outcome. The mediation analysis followed the causal steps approach and bootstrap procedures with 5,000 bootstrap samples to estimate direct effects (*X* → *Y*), indirect effects (*X* → *M* → *Y*), and total effects, with age, sex, education level, marital status, living arrangement, number of chronic conditions, and baseline scores of social participation, WHO-5 well-being, and GDS-15 depression included as covariates in all mediation models. Additional health outcomes analyzed included physical health measures assessed by the 6-Minute Walk Test (6MWT) (24) for aerobic endurance, Timed Up and Go (TUG) test for balance (25), Chair Sit-and-Reach Test for flexibility (26), and blood pressure measurements, as well as mental health measures including anxiety symptoms assessed by the Generalized Anxiety Disorder 7-item scale (GAD-7) (27) and health-related quality of life measured by the 12-Item Short Form Health Survey (SF-12) (28) yielding Physical Component Summary (PCS) and Mental Component Summary (MCS) scores. All primary analyses were conducted according to the intention-to-treat (ITT) principle, with all randomized participants included regardless of adherence or follow-up completion, using linear mixed-effects models with restricted maximum likelihood estimation to evaluate changes across assessment time points, with each model including fixed effects for group, time, and their interaction (Group × Time), baseline age, sex, and the baseline value of the outcome as covariates, participant ID modeled as a random intercept to account for repeated measurements, and community of recruitment included as a random effect to adjust for potential clustering. A per-protocol analysis including only participants who attended ≥80% of intervention sessions was performed as a sensitivity analysis to examine the robustness of findings among adherent participants, while additional sensitivity analyses examined dose–response relationships by analyzing the association between attendance rates (number of sessions attended or attendance proportion) and outcomes (social participation, WHO-5 well-being, GDS-15 depression, and other health measures), and subgroup analyses examined potential effect modification by sex and age stratum using interaction terms within the mixed-effects framework. Missing data patterns were evaluated, with mixed-model estimation under maximum likelihood used as the primary method for handling missing observations, as this approach uses all available data and is valid under missing-at-random assumptions, and additional multiple imputation procedures were conducted to confirm the robustness of findings. The analytical workflow proceeded in three stages. First, linear mixed-effects models under the intention-to-treat principle were used to examine overall intervention effects (Group × Time interaction) for primary and secondary outcomes across assessment points. Second, change scores (Δ values) were computed from baseline to post-intervention for mediation analyses. Third, mediation analyses were conducted using PROCESS Model 4 to examine whether post-intervention social participation statistically accounted for intervention-related changes in psychological outcomes, adjusting for relevant baseline covariates. This sequential approach allowed estimation of both overall treatment effects and potential indirect pathways. All analyses were performed using SPSS version 27.0 (IBM Corp., Armonk, NY, United States) with the PROCESS macro for mediation analyses, with statistical significance set at a two-sided *p*-value <0.05, and effect estimates presented as adjusted means with corresponding 95% confidence intervals, and standardized effect sizes (Cohen’s *d*) calculated for primary comparisons.

### 2.9 Ethical considerations

The study was conducted in accordance with the ethical principles of the Declaration of Helsinki and complied with all standards governing research involving human participants. Ethical approval was obtained from the Institutional Review Board of Zhengzhou Technology and Business University, China (Approval No.: IEC/ZTBU/SPE/759/2025-26). All participants received comprehensive information regarding the study objectives, procedures, potential risks, and their right to withdraw at any time without consequence, and provided written informed consent prior to enrollment. Participant confidentiality was maintained through the use of unique identification codes, and all data were securely stored with restricted access.

## 3 Results

Table 2 presents the baseline characteristics of the 150 participants, with 75 individuals allocated to the square dance group and 75 to the control group. The mean age of participants was comparable between the square dance group (70.77 ± 5.62 years) and the control group (69.85 ± 5.82 years). Baseline social participation scores were similar across groups, with mean values of 8.87 ± 2.09 in the square dance group and 8.16 ± 2.07 in the control group. Likewise, baseline subjective well-being measured by the WHO-5 showed nearly identical scores in the square dance (11.31 ± 2.28) and control groups (11.29 ± 2.23). Baseline depressive symptoms assessed using the GDS-15 were also comparable between groups, with mean scores of 6.43 ± 1.82 and 5.96 ± 1.53, respectively, indicating overall equivalence between groups prior to the intervention.

**Table 2:** Baseline characteristics of participants (*N* = 150).

Variable	Square dance (*n* = 75)	Control (*n* = 75)
Age (years), mean ± SD	70.77 ± 5.62	69.85 ± 5.82
Social participation (SP_T0)	8.87 ± 2.09	8.16 ± 2.07
WHO-5 well-being (T0)	11.31 ± 2.28	11.29 ± 2.23
GDS-15 depression (T0)	6.43 ± 1.82	5.96 ± 1.53

Table 3 summarizes pre–post changes in social participation, subjective well-being, and depressive symptoms across the square dance and control groups. Participants in the square dance group demonstrated a marked increase in social participation from baseline (8.87) to post-intervention (12.15), corresponding to a mean change of +3.28, whereas the control group showed only a modest increase (+0.83). Similarly, subjective well-being measured by the WHO-5 improved substantially in the square dance group, increasing from 11.31 at baseline to 17.04 post-intervention (Δ = +5.73), compared with a smaller improvement in the control group (Δ = +1.35). In contrast, depressive symptoms assessed by the GDS-15 decreased notably in the square dance group, with scores declining from 6.43 to 2.57 (Δ = −3.85), while the control group exhibited a relatively smaller reduction (Δ = −1.03). Overall, the table indicates greater improvements in social participation and well-being, along with larger reductions in depressive symptoms, among participants who engaged in the square dance intervention compared with controls.

**Table 3:** Pre-post changes in social participation, well-being, and depression.

Outcome	Group	T0 mean	T1 mean	Change (Δ)
Social participation	Square dance	8.87	12.15	+3.28
	Control	8.16	8.99	+0.83
WHO-5 well-being	Square dance	11.31	17.04	+5.73
	Control	11.29	12.64	+1.35
GDS-15 depression	Square dance	6.43	2.57	−3.85
	Control	5.96	4.93	−1.03

Table 4 presents the mediation analysis examining social participation as a mediator of the relationship between square dance participation and changes in subjective well-being using PROCESS Model 4. Square dance participation was a significant predictor of increased social participation (path a: *β* = 3.28, SE = 0.29, *p* < 0.001), and social participation was, in turn, significantly associated with improvements in WHO-5 well-being scores (path b: *β* = 0.89, SE = 0.08, *p* < 0.001). The total effect of square dance participation on changes in subjective well-being was significant (*β* = 5.73, SE = 0.42, *p* < 0.001). When social participation was included in the model, the direct effect remained significant but was reduced in magnitude (*β* = 2.85, SE = 0.26, *p* < 0.001), indicating partial mediation. The bootstrapped indirect effect (a × b = 2.92) was statistically significant, with the 95% confidence interval not including zero, confirming that social participation significantly mediated the effect of square dance participation on subjective well-being.

**Table 4:** Mediation analysis for subjective well-being (PROCESS model 4).

Path	*β*	SE	*p*-value
Square dance → Social participation (a)	3.28	0.29	<0.001
Social participation → ΔWHO-5 (b)	0.89	0.08	<0.001
Total effect (c)	5.73	0.42	<0.001
Direct effect (c′)	2.85	0.26	<0.001
Indirect effect (a × b)	2.92	Bootstrapped	95% CI ≠ 0

Table 5 presents the mediation analysis examining social participation as a mediator of the relationship between square dance participation and changes in depressive symptoms using PROCESS Model 4. Square dance participation was a significant predictor of increased social participation (path a: *β* = 3.28, SE = 0.29, *p* < 0.001), and higher social participation was significantly associated with reductions in depressive symptoms as measured by changes in GDS-15 scores (path b: *β* = −0.86, SE = 0.07, *p* < 0.001). The total effect of square dance participation on depressive symptoms was significant (*β* = −3.85, SE = 0.39, *p* < 0.001). After accounting for social participation, the direct effect remained statistically significant but was attenuated (*β* = −2.85, SE = 0.26, *p* < 0.001), indicating partial mediation. The bootstrapped indirect effect (a × b = −2.82) was statistically significant, with the 95% confidence interval not including zero, confirming that social participation significantly mediated the relationship between square dance participation and reductions in depressive symptoms.

**Table 5:** Mediation analysis for depressive symptoms (PROCESS model 4).

Path	*β*	SE	*p*-value
Square dance → Social participation (a)	3.28	0.29	<0.001
Social participation → ΔGDS-15 (b)	−0.86	0.07	<0.001
Total effect (c)	−3.85	0.39	<0.001
Direct effect (c′)	−2.85	0.26	<0.001
Indirect effect (a × b)	−2.82	Bootstrapped	95% CI ≠ 0

## 4 Discussion

The present study examined the mediating role of social participation in the relationship between square dance participation and well-being outcomes among older adults in China. The findings provide robust evidence that a 12-week square dance intervention significantly improved subjective well-being and reduced depressive symptoms, with social participation statistically mediating the association between the intervention and psychological outcomes. These results align with theoretical frameworks emphasizing the importance of social engagement as a mechanism through which physical activity influences mental health outcomes in older populations (17, 29).

The mediation analyses revealed that square dance participation exerted both direct and indirect effects on subjective well-being and depressive symptoms. The indirect pathway through social participation accounted for a substantial proportion of the total effect, demonstrating that the benefits of square dance extend beyond the physical activity itself to encompass meaningful social engagement. Specifically, participants in the square dance group demonstrated a mean increase of 3.28 points in social participation scores compared to controls, which subsequently predicted improvements in well-being (*β* = 0.89) and reductions in depressive symptoms (*β* = −0.86). These findings are consistent with socio-emotional selectivity theory, which posits that older adults prioritize emotionally meaningful social interactions that enhance well-being and life satisfaction (30, 31). Furthermore, the results support the convoy model of social relations, which emphasizes that supportive social networks serve as protective factors against age-related psychological decline (32).

The partial mediation observed in both models indicates that social participation explains a meaningful portion, but not all, of the intervention’s beneficial effects. The remaining direct effects (c′ = 2.85 for well-being; c′ = −2.85 for depression) suggest that additional mechanisms may be operative, including the physiological benefits of moderate-intensity physical activity (33), improvements in self-efficacy (34), enhanced cognitive function through learning choreographed movements (13), or the intrinsic enjoyment derived from musical and rhythmic engagement (35). This interpretation is consistent with previous research demonstrating that structured group-based exercise interventions yield multifaceted benefits for older adults through both biological and psychosocial pathways (36, 37).

The present findings corroborate and extend prior research on the mental health benefits of square dance participation among Chinese older adults. A systematic review and meta-analysis by Wu and colleagues (16) examining 23 studies found that square dance significantly improved depression symptoms (SMD = −0.89, 95% CI: −1.23 to −0.55) and quality of life among older adults, with effect sizes comparable to those observed in our study. Similarly, Ou and colleagues (38) conducted a randomized controlled trial with 120 older adults and reported that 12 weeks of square dance participation resulted in significant improvements in psychological well-being (*p* < 0.001) and reductions in loneliness scores, supporting our findings of enhanced subjective well-being.

Chen and colleagues (15) conducted a systematic review identifying social interaction as a key mechanism underlying the positive effects of square dance on psychological well-being, though few studies had formally tested mediation pathways. Our results provide empirical support for this hypothesized mechanism through rigorous mediation analysis with bootstrapped confidence intervals, strengthening causal inference regarding the role of social participation. Furthermore, Ou and colleagues (38) found that square dance participation increased social network size and frequency of social contact among community-dwelling older adults, which mediated improvements in mental health outcomes, directly paralleling our mediation findings.

The mediating role of social participation observed in our study aligns with substantial evidence linking social engagement to mental health in later life. A longitudinal study by Tomioka and colleagues (39) followed 7,770 older adults over 6 years and found that participation in group activities significantly predicted lower incidence of depressive symptoms, with social participation frequency showing a dose–response relationship with mental health outcomes. Similarly, Douglas and colleagues (40) demonstrated that older adults who engaged in group-based leisure activities reported higher life satisfaction and lower depression, with social connectedness serving as a key mediating variable. Our findings extend this literature by demonstrating that culturally specific physical activities can serve as effective vehicles for enhancing social participation and, consequently, psychological well-being.

Kanamori and colleagues (41) conducted a randomized controlled trial examining the effects of a group exercise program on social network development among older adults in Japan. Their results indicated that structured group activities facilitated the formation of new social connections, which subsequently predicted improvements in depressive symptoms (*β* = −0.32, *p* < 0.01), consistent with our mediation pathway. Additionally, Hwang and colleagues (42) found that frequency of social participation mediated the relationship between community-based physical activity programs and quality of life in Korean older adults, further supporting the generalizability of social participation as a critical mechanism across different cultural contexts. It is important to consider that the intervention group received structured group activity, regular social interaction, and facilitator attention, whereas the control group received health education materials only. Therefore, part of the observed benefits may reflect non-specific intervention components such as attention effects, expectancy effects, group belonging, or increased interpersonal contact rather than the unique characteristics of square dance itself. Future studies employing active control conditions, such as alternative group-based exercise or social programs, would help isolate the specific effects of square dance from broader social engagement effects.

The magnitude of improvement observed over the 12-week period was substantial. While clinically encouraging, such changes may partially reflect positive expectancy or novelty effects associated with participation in a culturally meaningful and socially engaging activity. Participants may have experienced enhanced motivation or reporting bias due to awareness of group allocation. Although outcome assessors were blinded, self-report measures remain susceptible to social desirability influences. Future studies incorporating objective behavioral or physiological indicators may help mitigate this limitation.

The magnitude of improvement in subjective well-being observed in the square dance group (Δ = +5.73 on the WHO-5) represents a clinically meaningful change, exceeding the minimal important difference threshold of approximately 10% improvement from baseline scores (43). Similarly, the reduction in depressive symptoms (Δ = −3.85 on the GDS-15) is substantial and clinically significant. According to established guidelines, a reduction of 2 points or more on the GDS-15 is considered clinically meaningful (44), indicating that our intervention achieved therapeutically relevant improvements. These effect sizes are comparable to or exceed those reported in meta-analyses of exercise interventions for depression in older adults. Schuch and colleagues (7) reported a pooled effect size of *g* = −0.90 (95% CI: −1.01 to −0.80) for exercise interventions on depression in older adults, while our study demonstrated an even larger between-group difference in depressive symptom reduction. Bridle and colleagues (2012) conducted a systematic review of 23 trials examining exercise interventions for depression in older adults and found moderate effect sizes (SMD = 0.34, 95% CI: 0.13–0.55), suggesting that culturally embedded group activities such as square dance may offer enhanced benefits compared to conventional exercise programs. The superior outcomes observed in our study may be attributable to the social and cultural dimensions of square dance, which integrates physical activity with music, group cohesion, and cultural identity (45).

The dual pathway through which square dance influences well-being, both directly through physical activity and indirectly through social participation, is supported by extensive literature. Bauman and colleagues (46) emphasized that the context in which physical activity occurs is as important as the activity itself, particularly for older adults who benefit from social interaction and group support. Smith and colleagues (47) found that older adults who participated in group-based exercise programs reported greater adherence and more substantial improvements in mental health compared to those engaging in individual exercise, highlighting the added value of the social component.

A meta-analysis by Pels and Kleinert (19) examining 38 studies concluded that group exercise programs produced larger effects on mental health outcomes compared to individual exercise interventions (*d* = 0.56 vs. *d* = 0.35), supporting the importance of social context. Our findings align with this evidence, demonstrating that the social participation fostered by square dance significantly contributes to psychological benefits beyond the effects of physical exertion alone. Furthermore, Withall and colleagues (48) reported that community-based group activities enhanced both objective social participation and subjective feelings of social connectedness, which collectively predicted improvements in mental health, mirroring the mechanisms identified in our mediation models.

Square dance represents a culturally meaningful activity deeply embedded in Chinese community life, which may enhance participation adherence and psychological benefits. Zhou and colleagues (49) noted that square dance functions as a form of social capital generation, fostering trust, reciprocity, and collective identity among participants. This cultural embeddedness distinguishes square dance from conventional exercise programs and may explain the robust improvements observed in our study. Wang and colleagues (50) found that older adults who participated in culturally congruent physical activities reported higher levels of intrinsic motivation and enjoyment, which are known predictors of sustained behavior change and psychological well-being.

Jaldin and colleagues (51) emphasized that square dance serves multiple functions beyond physical exercise, including opportunities for self-expression, cultural participation, and intergenerational connection, all of which contribute to a sense of purpose and belonging. Our findings suggest that interventions leveraging culturally specific practices may be particularly effective for promoting mental health in older adults, as they align with participants’ values, traditions, and social norms (52).

Although the primary analysis focused on intention-to-treat comparisons, the documented attendance rates provide insight into intervention feasibility and potential dose–response relationships. Previous research has established that higher frequency and longer duration of social participation are associated with greater mental health benefits. Tomioka and colleagues (39) found that older adults participating in group activities at least once per week had significantly lower rates of depression compared to those with less frequent participation, with the strongest effects observed among those attending multiple times per week. Similarly, Kanamori and colleagues (53) reported that attendance frequency in group exercise programs predicted the magnitude of improvement in depressive symptoms, with participants attending ≥80% of sessions demonstrating the largest benefits.

Our study protocol, which involved three 60-min sessions per week over 12 weeks, aligns with recommended guidelines for physical activity in older adults (54) and provides sufficient exposure to facilitate both physiological adaptations and social relationship development. Takeda and colleagues (55) noted that sustained participation over at least 8–12 weeks is necessary for meaningful changes in social network characteristics and mental health outcomes, supporting the duration of our intervention. While social participation emerged as a significant mediator, the persistence of direct effects in both mediation models suggests additional mechanisms warrant consideration. Neurobiological pathways may contribute to the observed benefits, as physical activity has been shown to increase brain-derived neurotrophic factor (BDNF), reduce inflammation, and modulate neurotransmitter systems involved in mood regulation (56, 57). Rehfeld and colleagues (13) demonstrated that dance training, which involves complex motor learning and spatial memory demands, produces greater increases in hippocampal volume compared to conventional endurance exercise, suggesting that the cognitive challenges inherent in learning choreographed routines may independently contribute to mental health improvements.

Additionally, the rhythmic and musical elements of square dance may activate reward pathways and enhance mood through emotional engagement with music (58). Dunbar and colleagues (59) proposed that synchronized movement and group cohesion trigger endorphin release, producing positive affective states and strengthening social bonds. These mechanisms may operate independently of social participation per se, contributing to the direct effects observed in our mediation models. Self-efficacy represents another plausible mechanism. McAuley and colleagues (34) found that improvements in physical self-efficacy mediated the relationship between exercise participation and mental health in older adults, suggesting that mastery of new skills and perceptions of physical competence contribute to psychological well-being. The progressive structure of our square dance intervention, which gradually increased complexity and duration over 12 weeks, likely facilitated self-efficacy development through successful performance experiences.

The findings have important implications for designing mental health promotion programs for older adults. First, the results suggest that community-based interventions leveraging culturally meaningful activities can serve as effective, low-cost strategies for enhancing well-being and reducing depressive symptoms. Square dance requires minimal equipment, can be conducted in public spaces, and naturally attracts participants through its cultural relevance and social appeal (45). Policymakers and public health practitioners should consider supporting and expanding access to such programs as part of comprehensive healthy aging initiatives. Second, the mediating role of social participation underscores the importance of designing interventions that explicitly facilitate social connection and community integration. Programs should prioritize group-based formats, create opportunities for interaction and relationship development, and foster inclusive environments that welcome diverse participants (48). Healthcare providers working with older adults experiencing social isolation or mental health difficulties might consider prescribing participation in community-based group activities as an adjunct or alternative to conventional treatments (60). Third, the findings highlight the potential for culturally adapted interventions to achieve superior outcomes compared to generic programs. Cross-cultural research has consistently demonstrated that interventions aligned with participants’ cultural values, preferences, and traditions achieve higher engagement and effectiveness (61). Public health efforts should prioritize the development and evaluation of culturally specific interventions tailored to local contexts and populations.

The study possesses several methodological strengths, including a randomized controlled design, blinded outcome assessment, formal mediation analysis with bootstrapped confidence intervals, comprehensive assessment of both mental health and social participation outcomes, and adequate statistical power to detect meaningful effects. The use of validated instruments (WHO-5, GDS-15) enhances the reliability and comparability of findings across studies. However, several limitations warrant acknowledgment. First, the sample size was relatively modest and participants were recruited from a single community center, which may limit external validity. Second, depressive symptoms were assessed using self-report measures, which may introduce response bias. Third, the 12-week intervention period, while sufficient to detect significant changes, may not capture long-term sustainability of benefits or the durability of social connections formed during the program. Future research should include extended follow-up periods to assess maintenance of effects. Fourth, self-report measures of social participation and mental health are subject to reporting bias, though the use of blinded assessors mitigates this concern. Fifth, although mediation analysis provides insight into potential causal pathways, the cross-sectional assessment of mediator and outcome at post-intervention limits definitive causal inference. Longitudinal mediation analyses with multiple time points would strengthen causal claims (62). Finally, while the study controlled for numerous covariates, unmeasured confounders such as personality traits, pre-existing social network characteristics, or concurrent life events may have influenced outcomes.

A key methodological limitation concerns the temporal ordering of the mediator and outcome variables. Social participation and psychological outcomes were assessed at the same post-intervention time point (Week 12). Although the statistical mediation model supports an indirect association, the design does not allow definitive conclusions regarding the directionality of effects. It remains possible that improvements in well-being encouraged greater social participation rather than social participation driving improvements in mental health. Future studies employing longitudinal mediation designs with multiple intermediate assessments are required to clarify causal sequencing. Future research should address these limitations through several approaches.

The predominantly female composition of the sample (approximately 85%) reflects the demographic characteristics of square dance participants in China but limits generalizability to older men. Gender differences in social engagement patterns, depressive symptom expression, and exercise preferences may influence intervention effects. Future studies should aim for more balanced gender representation to enhance external validity. First, multi-site studies encompassing diverse geographic regions, including rural areas, would enhance generalizability. Second, studies should actively recruit male participants to examine gender-specific effects and promote more balanced representation. Third, longitudinal designs with extended follow-up periods (e.g., 6–12 months post-intervention) are needed to assess the durability of mental health benefits and the stability of social relationships formed through square dance participation (63). Fourth, objective measures of physical activity (accelerometry) and social participation (ecological momentary assessment, social network analysis) would complement self-report data and provide more nuanced understanding of mechanisms (64).

Fifth, comparative effectiveness studies examining square dance against other forms of group exercise or social interventions would help identify active ingredients and optimal intervention characteristics (65). Sixth, qualitative research exploring participants’ subjective experiences, perceived mechanisms of benefit, and factors influencing adherence would provide valuable contextual understanding to complement quantitative findings (66). Seventh, investigation of moderating factors, such as baseline social isolation, depression severity, personality characteristics, or cultural identity, could inform personalized intervention strategies and identify subgroups most likely to benefit. Finally, cost-effectiveness analyses comparing square dance interventions to alternative mental health promotion strategies would inform resource allocation decisions and policy development (67).

## 5 Conclusion

This randomized controlled trial provides robust evidence that square dance participation significantly improves subjective well-being and reduces depressive symptoms among community-dwelling older adults, with social participation statistically accounting for part of the association between square dance participation and psychological outcomes. The findings demonstrate that culturally embedded group physical activities can serve as effective, accessible interventions for promoting mental health in aging populations. Both direct pathways, through physiological, cognitive, and affective benefits of physical activity and indirect pathways, through enhanced social engagement and community integration, contribute to the observed improvements. These results support the integration of culturally meaningful group activities into comprehensive mental health promotion strategies for older adults and highlight the importance of social participation as a modifiable target for intervention. Future research should examine long-term sustainability, explore additional mediating mechanisms, and evaluate implementation in diverse populations and settings to maximize the public health impact of these promising findings.

## Data Availability

The original contributions presented in the study are included in the article/Supplementary material, further inquiries can be directed to the corresponding author.
